# Enhancing Linearity of Light Response in Avalanche Photodiodes by Suppressing Electrode Size Effect

**DOI:** 10.3390/s24113366

**Published:** 2024-05-24

**Authors:** Hongyi Gan, Junwen Yu, Xiangfu Wang

**Affiliations:** 1 College of Electronic and Optical Engineering & College of Flexible Electronics (Future Technology), Nanjing University of Posts and Telecommunications, Nanjing 210023, China; b21020308@njupt.edu.cn (H.G.); 1022020601@njupt.edu.cn (J.Y.); 2 The State Key Laboratory of Refractories and Metallurgy, Wuhan University of Science and Technology, Wuhan 430081, China

**Keywords:** avalanche photodiodes, linearity, electrode, gallium nitride

## Abstract

The nonlinear characteristics of avalanche photodiodes (APDs) inhibit their performance in high-speed communication systems, thereby limiting their widespread application as optical detectors. Existing theoretical models have not fully elucidated complex phenomena encountered in actual device structures. In this study, actual APD structures exhibiting lower linearity than their ideal counterparts were revealed. Simulation analysis and physical inference based on GaN APDs reveal that electrode size is a noteworthy factor influencing response linearity. This discovery expands the nonlinear theory of APDs, suggesting that APD linearity can be enhanced by suppressing the electrode size effect. A physical model was developed to explain this phenomenon, which is attributed to charge accumulation at the edge of the contact layer. Therefore, we proposed an improved APD design that incorporates an additional gap layer and a buffer layer to stabilize the internal gain under high-current-density conditions, thereby enhancing linearity. Our improved APD design increases the linear threshold for optical input power by 4.46 times. This study not only refines the theoretical model for APD linearity but also opens new pathways for improving the linearity of high-speed optoelectronic detectors.

## 1. Introduction

Avalanche photodiodes (APDs) are highly sensitive photodetectors with a high gain, which are widely used in optical communication, light detection, imaging, and other fields [1,2,3]. Compared with optical receivers comprising other optoelectronic devices, APDs offer unique advantages in terms of signal sensitivity and signal transmission distance in optical communication systems because of their internal gain [4,5,6]. However, the presence of these advantages also brings high current density and low linearity [7]. The low linearity of APDs restricts their usage in communication systems exceeding 10 Gbit/s [8,9,10]. Some studies suggest that highly linear APDs can extend transmission distances while maintaining low power consumption [11] and enhancing the sensor performance [12,13]. GaN-based APDs have garnered significant attention for their outstanding potential in high-sensitivity visible light or solar-blind detection. This is because GaN-based APDs can eliminate the need for expensive optical filters and considerably improve system integration and reliability. Recently, some sun-blind UV communication systems have also been proposed [14]. GaN-based APDs show the potential to achieve bandwidths as high as 62.9 GHz [15], which offers the potential advantage of high bandwidth and demonstrates a unique benefit for application in optical communication systems.

**Figure 1:**
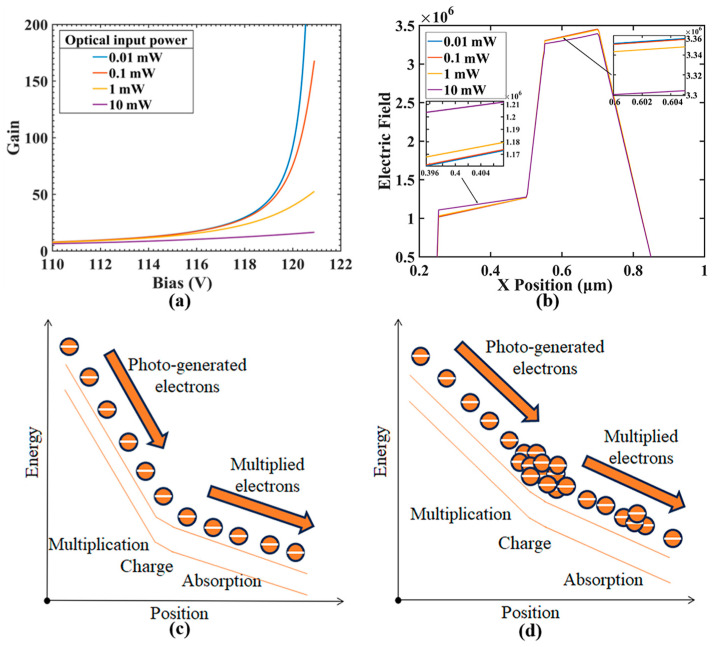
(**a**) Relationship between avalanche gain (gain) and bias voltage under different optical input powers. (**b**) Cross-section electric field at different optical input powers. The band diagram of the separated absorption and multiplication regions of APDs when the optical input is (**c**) low and (**d**) high, suggesting the accumulation of space charges at the charge layer.

Although early research extensively analyzed the nonlinear phenomena in APDs and provided theoretical explanations applicable to one dimension [7,16], the discovered new phenomenon–lower linearity in the actual structure of APDs than ideal structures–remains unexplained by existing theories; the difference between the ideal and actual structures used in simulation can be found in the next section. The nonlinearity in APDs manifests as a degradation of internal gain with increasing optical input power, thereby decreasing light response (Figure 1a,b), a phenomenon that was deeply analyzed in detail in earlier studies [7,16]. The principle involves the space charge effect at the charge layer (Figure 1c,d). With the saturation of the internal current, a large number of carriers accumulate at the end of the depletion layer, forming space charges that neutralize the depletion layer’s charges, resulting in a decrease in the electric field intensity in the multiplication region and a reduction in the impact ionization rate. Subsequent studies have analyzed this principle and the issue of linearity improvement [17,18,19], but mainly within one-dimensional (1D) ideal scenarios; thus, in-depth explorations regarding more complex situations or comparative analyses are lacking.

**Figure 2:**
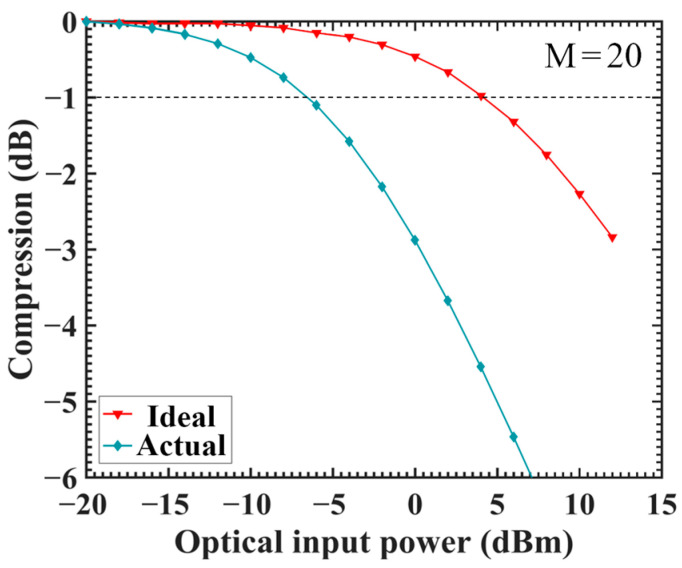
Comparison of linearity between the ideal and actual structures under an initial multiplication gain of 20 (namely, APD bias). Y axis denotes the compression of the optical response and the initial gain of the two lines is the same (namely, APD bias).

We compared the linearity differences between APDs closely resembling actual and ideal structures via simulations and revealed a remarkable decrease in linearity for APDs close to the actual device (Figure 2). The P1dB compression point was used to compare the linearity differences. Compression refers to a reduction in the actual optical response compared with the perfectly linear light response, whose formula is given by Equation (1). The optical input power corresponding to the −1 point of compression is the optical input power threshold, i.e., P1dB or −1 dB [8,17]. Briefly, when the compression of the optical response (Y axis) is less than −1, the device is considered to remain linear.

(1) $$\mathrm{Compression}=10\log\left(\frac{R_{opt}}{R_{opt\_ideal}}\right)$$

$R_{opt}$ is the actual optical response, defined as the ratio of the optical current to the incident light power. And $R_{opt\_ideal}$ is the ideal optical response, which is the expected response under perfectly linear conditions. Here, we consider that a response with an optical input power of 0.01 mW is the ideal optical response. A smaller compression value indicates a larger deviation of the actual response from the ideal response, signifying a lower linearity of the device. The unit of optical input power is expressed in dBm.

Figure 2 shows that the P1dB compression points for the ideal and actual devices are 4.0 and −6.5 dBm, respectively. This means that the optical input power threshold of APDs close to the actual application is reduced by 11.2 times (from 2.511 to 0.224 mW). Because the thicknesses and doping concentrations of each layer in the ideal and actual structures are consistent, existing theories fail to explain this phenomenon. To further understand the underlying mechanism behind this phenomenon, we performed simulations and physical analyses based on a two-dimensional model, and the results revealed that a phenomenon referred to as the “electrode size effect”, which is not only widely recognized but is prevalent, is a crucial factor leading to a decay in device linearity.

The effect of the electrode size on APDs was first analyzed by Yang et al. [20], suggesting that the gain will decrease with the decreasing electrode size, which is believed to be caused by the current-crowding effect induced by resistance. We can observe by adjusting the electrode size (in Section 3.1) that this effect on the gain associated with the electrode size increases with the increasing incident light power (alternatively, an increase in optical input power amplifies the magnitude of the gain decrease). Thus, the effect of the electrode size can be extended to the linearity (a low linearity is defined as the gain decreases with the increasing optical input power, and our result in Section 3.1 shows that the effect of the electrode size on thr gain increases with increasing optical input power). However, the explanation provided by Yang et al. [20] for the observed effect seems inadequate. Compared to the resistance, this appears to be more likely due to the space charge influence of the multiplication layer electric field. Our simulation results suggest that this phenomenon is also not entirely consistent with the current-crowding effect. To further clarify these observations, we propose a physical model based on the space charge theory to elucidate the impact of electrode size on the device performance.

In some previous studies, electrodes were considered a relatively minor factor, with differences in shape and size even being ignored. In this study, we examine a relatively common electrode shape and we believe that this common electrode shape induces a physical process, as illustrated in Section 3.4, resulting in a change in gain, electric field, and linearity. However, practical designs often feature various specialized electrode shapes [21,22], and devices incorporating such electrodes may not fully adhere to the physical process depicted in Section 3.4. The alteration in such a physical process may impact these devices’ properties. Nevertheless, these studies did not indicate whether the specialized electrodes could impact device properties [21,22]. Our findings suggest that investigating the effects of these specialized electrode designs on the trajectory of charge carriers and the overall electric field strength of the device is crucial.

Similarly, our findings demonstrate that employing 1D simulation and neglecting differences in electrode size may lead to linearity overestimation. This situation occurred in some studies that used simulation methods [17,18,19]. These simulation methods adopt a 1D simulation approach, in which a three-dimensional structure is abstracted into a 1D line, thereby overlooking lateral variations in the device. Our comparison of the ideal and actual devices (Figure 2) suggests that the 1D approach introduces errors when simulating device properties. Thus, these designs, validated by simulation programs, may fail to achieve the claimed high linearity in actual situations. If the underlying physical processes behind the effect of electrodes on device properties are thoroughly explored, these studies on linearity could lead to significant improvements.

In this study, we observe that device geometries that more closely resemble realistic experimental devices exhibit lower linearity compared to idealized 1D structures. We performed a comparative analysis to examine the correlation between structural differences and linearity. Through simulation studies, we determined the electrode size effect on linearity reduction, thereby offering a further theoretical explanation for the nonlinear characteristics of APDs. For the first time, we propose a physical model that provides an in-depth explanation regarding the effect of the electrode size on APD linearity reduction. Additionally, we suggest an innovative design for APDs that can suppress the electrode size effect to improve linearity. This is done by incorporating the gap and buffer layer structure, which mitigates the effect of the electrode size on the space charge accumulation, thereby maintaining the stability of internal gain at high currents and enhancing device linearity.

## 2. Materials and Methods

**Figure 3:**
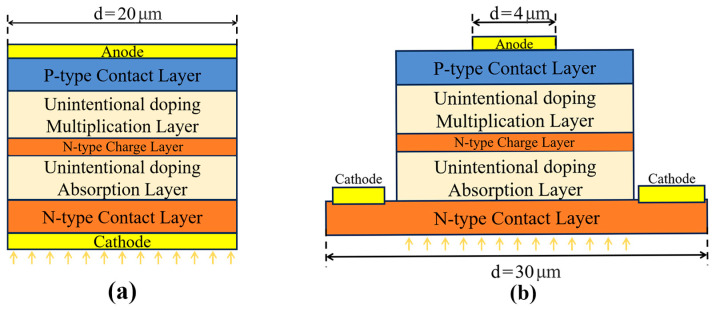
(**a**) The ideal structure avalanche photodiode. (**b**) The actual structure avalanche photodiode.

A series of GaN-based separated absorption and multiplication region APDs (SAM-APDs) were established for simulation purposes. Figure 3 illustrates the fundamental structure of these APDs utilized in the simulation, with a device diameter of 20 μm and uniform vertical structural characteristics. Ideally, APDs are abstracted into a 1D idealized model by neglecting the transverse difference of the device. However, in the actual design, the top electrode size is usually smaller than the top mesa and the bottom electrode is placed on the bottom mesa (here, it refers to the bottom contact layer). It is important to emphasize that the ideal structure considered in our previous discussion is inherently 1D. However, for comparative analysis, the idealized structure used in the simulations merely represents a simplification of the actual structure. In the ideal structure, incident light continues to ingress from the bottom region, with adjustments made to the transmission coefficient of the cathode.

The specific structural parameters are referenced in Table 1, and the design and parameters are drawn from previous work [23]. Further in the text, our study proceeds by manipulating the top Anode size, the n-type contact layer size, and the Cathode position.

The steady-state simulation was set up following the drift–diffusion method [24], which encompasses the Poisson equation and carrier continuity equations. In the carrier drift process, the generation–recombination process, the migration process, the collision ionization process, and the tunneling process will be used to consider their effects. The generation–recombination model utilized in this simulation includes a Radiative, a Shockley–Read–Hall (SRH), an Auger, a generation-recombination model, a mobility model that includes field dependent mobility, and a concentration-dependent mobility model; the tunneling model includes a band-to-band Tunneling model. Moreover, the impact of velocity saturation on mobility due to high electric fields is also taken into consideration. The parameters used in the simulation are listed in Table A1. The following formula describes the carrier generation–recombination model. The specific calculation formulas are listed in the Appendix A Equations (A1)–(A12).

## 3. Results and Discussion

### 3.1. What Reduces the Linearity

The traditional theory of the space charge within the charge layer applies only to 1D conditions. Through comparative simulations, it has been observed that actual APD structures exhibit lower linear response characteristics than the ideal models, as demonstrated in Figure 2. Given that the thickness and doping concentration of each layer in the ideal and actual structures are the same in the experiment, traditional theories fail to fully account for this phenomenon. To thoroughly understand the fundamental reasons behind this disparity, we divided the structural differences between actual and ideal cases into three parts and conducted separate simulation studies for each: (1) Bottom contact layer dimensions. (2) Cathode placement. (3) Anode dimensions

**Figure 4:**
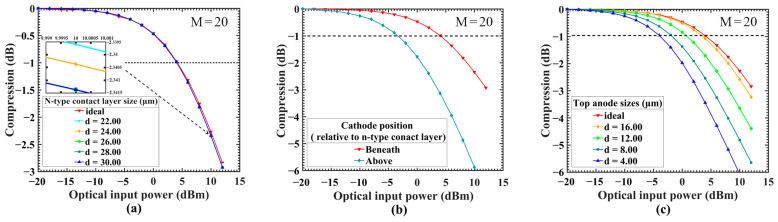
(**a**) Comparison of the linearity of devices with different N-type contact layer sizes in a device structure with d = 30 μm and the initial gain of 20, the same as below. (**b**) A comparison of the linearity of designs where the cathode is placed above and beneath the contact layer. (**c**) A comparison of the linearity of devices with different anode sizes.

By adjusting the dimensions of the bottom contact layer, the results obtained are shown in Figure 4a: The electrode is kept under the contact layer, after adjusting the size of the contact layer and the change to the P1dB compression point is less than 0.001 dBm, which can be almost ignored. The apparent change can be observed after the optical input power reaches 10 dBm (small figure in the upper right corner). By moving the cathode position, the result is shown in Figure 4b: When the electrode is placed above the n-type contact layer at d = 30 μm, the P1dB is reduced by nearly 7.5 dBm. Adjustments to the size of the top electrode are presented in Figure 4c: When the electrode size is diminished to 16um, the linearity begins to decline. As the electrode size gradually reduces, the P1dB decreases from the initial 4.0 dBm to −4.0 dBm. This suggests that the effect of the electrode size on the gain increases with the incident optical power. By comparing these results, we can see that the main factors that affect the reduction in linearity are the location and size of the electrodes. The effect of the mesa size of the n-type contact layer is not apparent and it is the electrodes that contribute most significantly to the low linearity.

**Figure 5:**
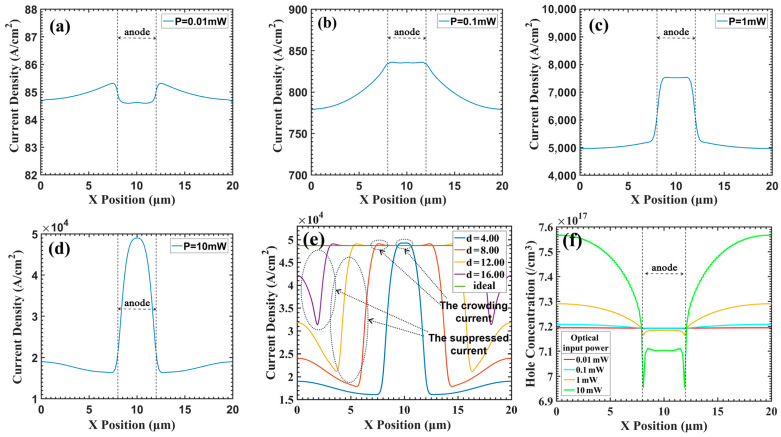
The cross-section of current density along X position between different optical input power (**a**) 0.01 mW (**b**) 0.1 mW (**c**) 1 mW (**d**) 10 mW, when the APDs bias are the same (the gain of (**a**) is 20), and (**e**) represents the comparison of current density across the cross-section for different electrode sizes when the optical input power is set to 10 mW The figure is symmetrical; hence, the label does not affect readability. The cross-section is located at a depth of 0.18 μm below the anode. (**f**) represents the hole concentration profile at a cross-section about 0.12 μm below the anode.

Yang et al. attributed the effect of the electrode size on the gain to the current-crowding effect [20]. From simulations that recorded electric currents, we also observed a similar phenomenon of current crowding after the reduction in electrode size, as shown in Figure 5a–d. Ideally, the internal current density of the device is considered to be uniformly distributed when the electrode size is reduced, and the current only retains high intensity in the central region—this is what is known as current crowding [20].

However, Figure 5e shows that the crowding current is not evident. Only an extremely small portion of the current is concentrated in the center (the arrow in Figure 5e points this out). The reason for the decrease in the device gain is more likely that the current in the edge region is suppressed. To further explore the essence of the electrode effect and give a reasonable theoretical explanation of the physical mechanism of the phenomenon, we have developed a new physical model. We recorded changes in various physical quantities during the simulation process, including the distribution of the electric field along the X position, the current density, and the hole concentration. These simulation results helped us uncover the physical mechanisms underlying the “current crowding” formation. Based on these insights, we constructed a comprehensive physical description framework aimed at systematically elucidating the mechanisms of electrode size effect formation.

### 3.2. What Causes the Electrode Size Effect

**Figure 6:**
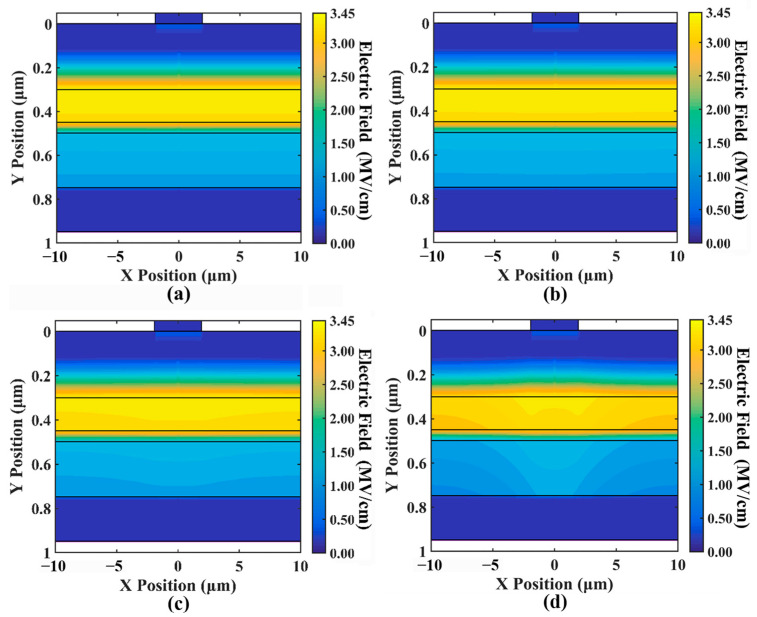
(**a**–**d**) show the electric field intensity distribution for different optical input powers of 0.01 mW, 0.1 mW, 1 mW, and 10 mW, respectively, with an initial gain of 20; the same below.

Figure 6 shows the distribution of the electric field inside the device. From the figure, we can see that with the increase in optical input power (the current increases), the electric field at the edge of the device gradually decreases. The field strength at the most affected point is reduced by more than 0.5 MV/cm. Due to the exponential relationship between the collision ionization coefficient and the electric field intensity (the specific formula can be found in Equations (A10) and (A11), this decrease in electric field intensity will significantly reduce the avalanche ionization rate, significantly reducing multiplication gain. However, the change in the electric field strength near the center of the device is negligible. This is the main reason for the low current at the edge of the device and the high current near the center.

**Figure 7:**
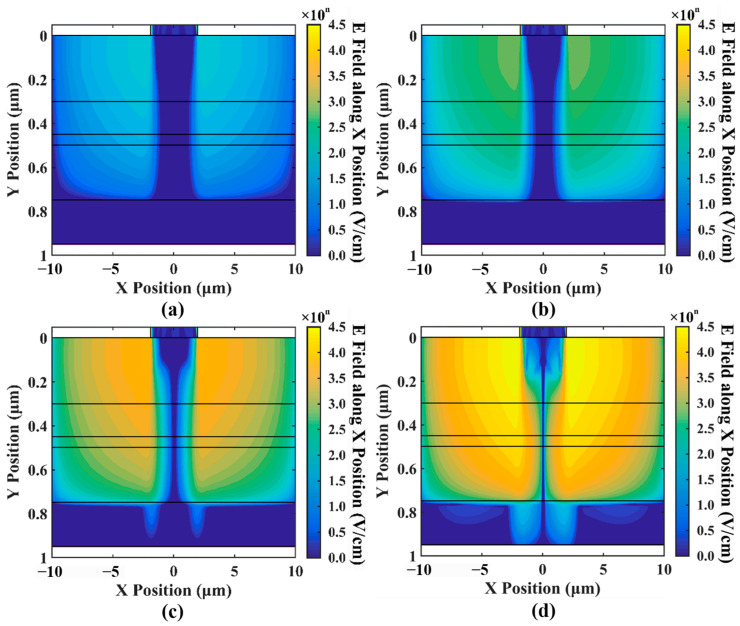
(**a**–**d**) show the electric field along the X position intensity (with units differing from the former) for different optical input powers of 0.01 mW, 0.1 mW, 1 mW, and 10 mW.

Figure 7 shows the distribution of the electric field along the X position (i.e., lateral electric field). As shown in the figure, the electric field along the X position, which should not exist, increases exponentially as the current increases. Previous 1D simulations have typically implied an assumption that there are no significant lateral factors within the device that can influence its properties. However, our simulations indicate the presence of the electric field along the X position within the device.

When the optical input power is 10 mW, the electric field along the X position even reaches about $4\times 10^{4}$ V/cm ($\mathrm{or}\;1\times 10^{4.6}\;V/\mathrm{cm}$). When it increases to a certain level, it can cause the carriers to converge towards the center gradually, which to some extent leads to current crowding in Figure 5. In addition, it is worth noting that the magnitude of the increase in the electric field along the X position is close to the magnitude of the increase in the optical input power, as shown in Figure 7; for every order of magnitude increase in the optical input power, the electric field along the X position also increases by an order of magnitude. Based on this, we speculate that there is a strong correlation between the two.

**Figure 8:**
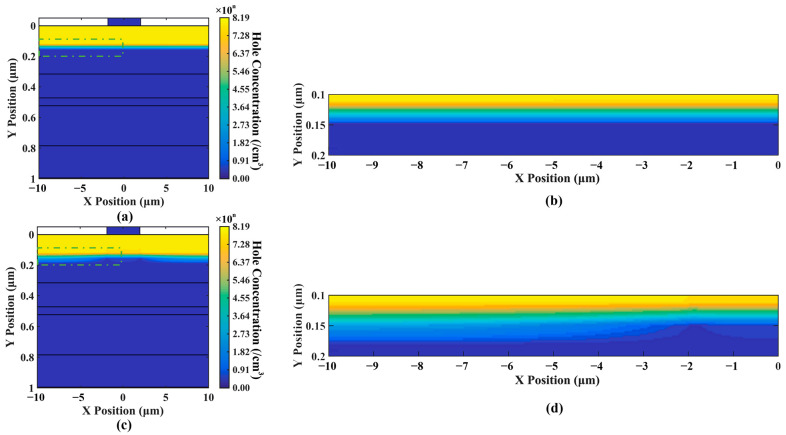
(**a**,**c**) display hole concentration distribution for different optical input powers of 0.01 mW and 10 mW. (**b**,**d**) depict enlargements of the regions outlined by the green dashed boxes in (**a**,**c**), respectively. Higher optical input power leads to non-uniform carrier concentration.

We believe that the variation in the electric field intensity and its X-directional component is caused by the accumulation of charge at the p-type contact layer, as shown in Figure 5f and Figure 8. For Figure 8a, it can be observed that the carrier (in this case holes) concentration along the black dashed line distribution is essentially uniform. However, for Figure 8b, it is evident that close to the black dashed line the hole concentration near the edge regions is significantly higher than in other regions. Moreover, as depicted in Figure 5f, it can be observed that with the increase in optical input power, the difference in carrier concentration between the edge and the center also increases. Due to the absence of anode coverage at the top edge of the p-type contact layer, the multiplied carriers cannot leave the device directly upon reaching the p-type contact layer; they must turn and pass through the anode at the center of the device to exit. This makes the carrier path at the edge region longer than that in the center region, resulting in the carriers at the edge leaving the device later than those at the center. This ultimately leads to an accumulation of carriers at the edge of the p-type contact layer, affecting the electric field distribution. This implies that the effect of the electrode size is essentially a manifestation of space charge effects, wherein space charge accumulates at the edges of the contact layer.

### 3.3. A Model for Interpretation

**Figure 9:**
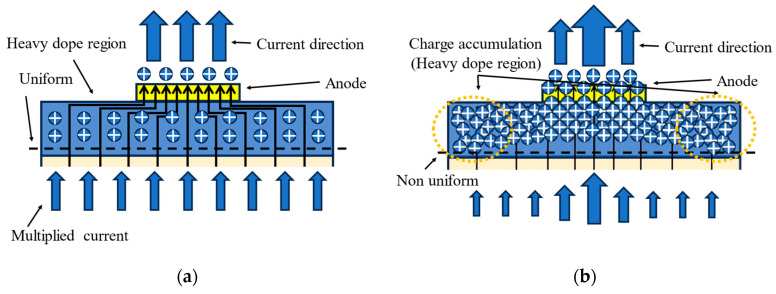
Shows the cross-section of the charge distribution when the optical input power is low (**a**) and high (**b**). (**a**) When the light power is low, the current sequentially leave the p-type contact layer, and no stable space charge is generated. (**b**) When the light power is high, the charge accumulates at the edge of the contact region. At this time, the current in the edge region is suppressed.

We have developed a physical model to explain this phenomenon more intuitively. As shown in Figure 9, when the current is low, the carriers migrate in order in the p-type contact layer and do not create significant spatial charge accumulation. However, when the current increases, the carriers at the center leave the electric field faster than those at the edge. For the same group of carriers, when the carriers at the center have already migrated out of the electric field, the carriers at the edge are still undergoing lateral migration, resulting in non-uniform carrier concentrations. This non-uniform concentration creates a space charge at the edge of the p-type contact layer, which then disrupts the electric field distribution, ultimately altering the electrical characteristics of the device.

Compared to previous theories, our research suggests that under high optical input power, the accumulation of space charge may not be limited to the charge layer but could also occur at the edge of the contact layer. This observation significantly enriches the theoretical framework regarding the nonlinearity of APDs. Additionally, in multi-junction structured APDs and certain specific configurations [25], similar charge accumulation phenomena may occur at the mesa’s edge, where differences in mesa size may cause local carrier accumulation, thereby affecting the device’s electrical characteristics.

Moreover, in-depth analysis is necessary to understand the unique characteristics of three-dimensional complex structures, such as Schottky electrodes or floating guard rings. Further discussion is also needed to determine whether simpler structures, such as the two-dimensional structures mentioned in [26], can improve linearity. The emergence of this phenomenon highlights the need for a comprehensive analysis of charge accumulation behavior in complex or uniquely structured APDs. Thus, future work will require more refined experimental designs and theoretical studies to evaluate comprehensively the specific impact of differential junction sizes and other unique structural features on the electrical performance of the devices.

### 3.4. Verification on Ring Electrodes

In addition to the round electrodes utilized in the aforementioned simulations, ring electrodes are also a commonly encountered electrode configuration [27,28]. To validate whether our theory is applicable to ring electrodes, we modified the electrode structure used in the simulations to incorporate two types of ring electrodes. The results are depicted in the following figure.

**Figure 10:**
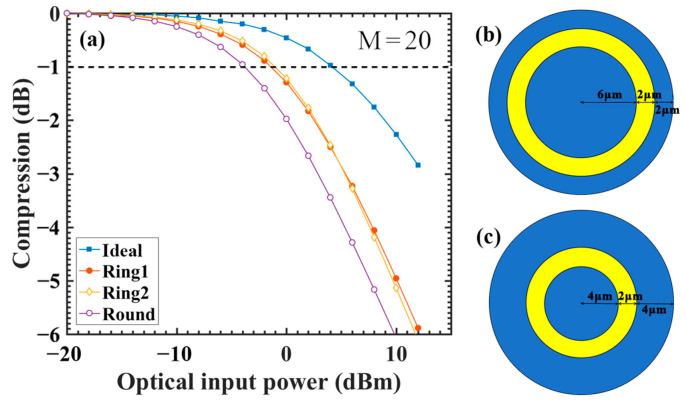
(**a**) Comparison of linearity between the ideal, the round electrode with d = 4 μm, and the two kinds of ring electrode structure devices under the initial gain of 20. (**b**) The structure of Ring 1. (**c**) The structure of Ring 2.

Figure 10 demonstrates that, compared to round electrodes, the linearity of ring electrodes exhibits a slight improvement. However, compared to the ideal scenario, there remains a significant decrease in linearity. This suggests that the space charge effect continues to cause a reduction in device linearity under ring electrode configurations.

### 3.5. Improved Design

**Figure 11:**
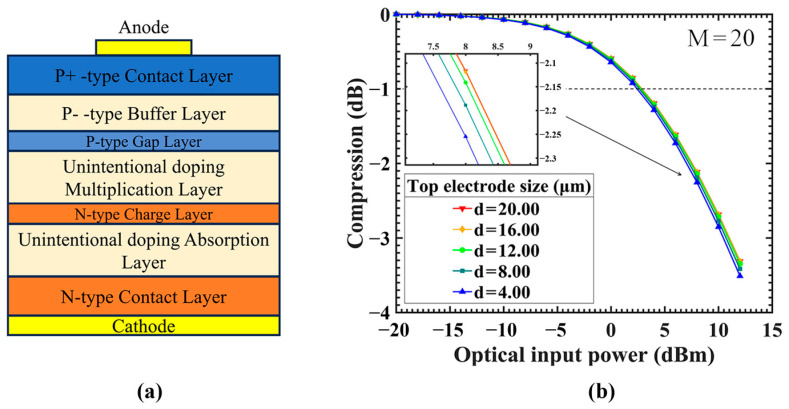
(**a**) Improved design of APD with an additional gap layer and a buffer layer. (**b**) The comparison of linearity between different anode sizes; the initial gain is 20. The linearity of APDs has effectively mitigated the impact of the electrode size.

In order to prevent the accumulation of charge at the edges of the contact layer from interfering with the electric field in the multiplicative region, we propose an improved design as shown in Figure 11a. To shield the electric field disturbances caused by the accumulated charge, we introduce a buffer layer with a doping concentration of $3\times 10^{17}$ cm^−1^ beneath the p-type contact layer, with a thickness of 0.25 μm. Additionally, we embed a 0.05 μm thick gap layer with a doping concentration of $5\times 10^{18}$ cm^−1^ above the multiplication region to ensure the stability of the electric field in the multiplication region. The doping concentration of the contact layer is increased to $5\times 10^{18}$ cm^−1^.

Figure 11b shows the linearity results after adjusting the size of the anode. The linearity is not significantly affected by the electrode size, and the effect of charge accumulation at the edge of the mesa (electrode size effect) is basically eliminated. The compression points of P1dB are concentrated around 2.5 dBm. Compared to the standard design with the same electrode size shown in Figure 4c, it is −4.0 dBm. Although, compared with Figure 4c, the linearity is slightly lower when the prepared electrode size is greater than 12 μm. If small-size electrodes are not needed, the device itself maintains good linearity, so there is no need to improve the design.

**Figure 12:**
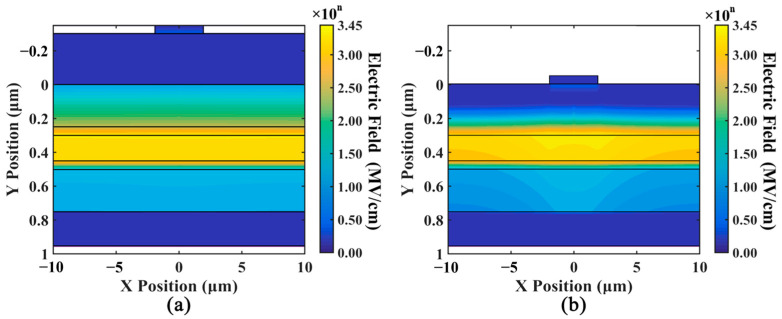
The comparison of the electric field between (**a**) improved design and (**b**) standard structure when the optical input power is 10 mW. The diameter of the anode is 4 μm.

Figure 12a shows the distribution of the internal electric field in the improved device design, compared to the traditional structure in Figure 12b. Under high optical input power conditions, the electric field distribution in the edge region of the improved design remains stable. Compared to traditional structures, the improved design reduces the disparity in the electric field between the central and edge regions.

**Figure 13:**
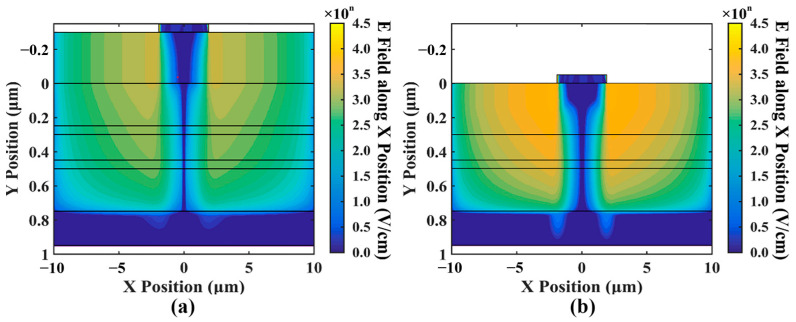
The comparison of the electric field along the X position between (**a**) the new design and (**b**) the standard structure when optical input power is 10 mW. The diameter of the anode is 4 μm.

The distribution of the electric field along the X position in the improved device design is shown in Figure 13a, compared to the traditional structure in Figure 13b. We can observe that the electric field along the X position in the new design is significantly lower than that in the traditional design. The electric field along the X position originates from the difference in carrier concentrations between the central and edge regions. The reduced electric field along the X position suggests that our design not only increases the distance between the accumulation charge and the multiplication layer but also reduces the carrier accumulation at the edges of the p-type contact layer.

## 4. Conclusions

We have identified the electrode size as a novel factor influencing the linearity of avalanche photodiodes (APDs). The essence of the electrode size effect lies in the accumulation of space charge at the edge of the contact layer. This accumulation can cause the electric field strength in the multiplicative region to decrease at high currents, leading to a degradation of the gain at the edges. To suppress this effect and enhance linearity, we propose to insert a buffer layer and a gap layer beneath the contact layer. This approach is intended to mitigate the effect of the space charge on the multiplicative layer. Under the condition of a 4-micron electrode diameter, the improved design significantly improves linearity, raising the P1dB compression point from −4.0 dBm to 2.5 dBm, corresponding to an increase in the optical input power threshold by approximately 4.46 times (0.398 mW to 1.777 mW). Our work contributes to enhancing the theoretical model for APD linearity and paves the way for improving the linearity of the device.

## Figures and Tables

**Table 1:** The parameters of the structure.

	Contact Layer	Multiplication Layer	Gap Layer	Absorption Layer	Contact Layer
Thickness (μm)	0.3	0.15	0.05	0.25	0.2
Doping (cm^−3^)	1 $\times \;10^{18}$	5 $\times \;10^{16}$	2 $\times \;10^{18}$	5 $\times \;10^{16}$	5 $\times \;10^{18}$

## Data Availability

Data are contained within the article.

## Appendix A

Here are the Poisson equation and carrier continuity equations.

(A1) $$\frac{\partial p}{\partial t}=-\frac{1}{q}\nabla {\vec{J}}_{p}+G-R$$

(A2) $$\frac{\partial n}{\partial t}=-\frac{1}{q}\nabla {\vec{J}}_{n}+G-R$$

(A3) $$\nabla^{2}\psi =-\frac{1}{\varepsilon_{0}\varepsilon }\left(\Gamma +p-n\right)+\frac{1}{\varepsilon }\nabla \psi \nabla \varepsilon$$

n, p is carrier concentration, *q* is electronic electricity, G is the carrier generation rate, *R* is the carrier recombination rate, Γ effective doping concentration, ε is the relative dielectric constant, $\varepsilon_{0}$ is the vacuum dielectric constant of the material. The optical generation rates of carriers are calculated using Equation (A4).

(A4) $$G^{opt}\left(z\right)=J\left(x,y,z_{0}\right)\cdot \alpha_{opt}\left(\lambda ,z\right)\cdot exp\left[-\left|\int_{z_{0}}^{z}\alpha \left(\lambda ,z\right)dz\right|\right]$$

where λ is the wavelength, $J\left(x,\;y,\;z_{0}\right)$ is the spatial distribution of optical beam intensity, and $\alpha_{opt}$ is the absorption coefficient of GaN. The recombination rate of the SRH model [29] can be calculated by Equation (A5).

(A5) $$R^{SRH}=\frac{pn-n_{i}^{2}}{\tau_{n}^{SRH}\left[n+n_{i}exp\left(\frac{\Delta E^{trap}}{kT_{L}}\right)\right]+\tau_{p}^{SRH}\left[p+n_{i}exp\left(\frac{-\Delta E^{trap}}{kT_{L}}\right)\right]}$$

$\Delta E^{trap}$ represents the difference between the trap energy level and the intrinsic Fermi level, $\tau_{n,p}^{SRH}$ is the lifetimes of carriers, where $n_{i}$ is the intrinsic carrier concentration and is given for Boltzmann statistics [30] by Equation (A6).

(A6) $$n_{i}=\sqrt[{2}]{N_{c}N_{v}}exp\left(\frac{-E_{g}}{2kT}\right)$$

$N_{c,v}$ is the effective density of states in the conduction and valence bands, $E_{g}$ is bandgap energy, k is the Boltzmann constant, and T is the absolute temperature. The recombination rate of the Auger model [31] can be calculated by Equation (A7).

(A7) $$R^{Auger}=C_{n}^{Auger}\left(pn^{2}-nn_{i}^{2}\right)+C_{p}^{Auger}\left(p^{2}n-pn_{i}^{2}\right)$$

$C_{n}^{Auger}$ and $C_{p}^{Auger}$ are Auger coefficients. The carrier generation rate due to band-to-band tunneling [32] is calculated using Equation (A8).

(A8) $$R^{BBT}=A_{BBT}E^{\gamma_{BBT}}exp\left(-\frac{B_{BBT}}{E}\right)$$

$A_{BBT}$, $\;B_{BBT}$, and $\gamma_{BBT}$ are BBT coefficients, and E is the electric field. The impact generation rate is calculated using Equation (A9) from [33].

(A9) $$G_{impact}=\alpha nv_{n}+\beta pv_{p}$$

α,β are the electron and hole ionization coefficients [34], calculated by

(A10) $$\alpha \left(E\right)=a_{n}exp\left[-{\left(\frac{b_{n}}{E}\right)}^{\gamma }\right]$$

(A11) $$\beta \left(E\right)=a_{p}exp\left[-{\left(\frac{b_{p}}{E}\right)}^{\gamma }\right]$$

$a_{n,p}$, $b_{n,p}$ are fitting coefficients, these coefficients come from [35]. Here we take γ as 1. Additionally, the avalanche gain mentioned later is calculated using Equation (A12).

(A12) $$M=\frac{I_{light}-I_{dark}}{I_{light0}-I_{dark0}}$$

$I_{light}$, $I_{dark}$ are the light and dark current, and $I_{light0}$, $I_{dark0}$ are the light and dark current that have not been multiplied; for some kinds of GaN-based APDs, it can be the initial light and dark current.

**Table A1:** The parameters for simulation.

Parameters (GaN)	Units	Electron	Hole
$\mathrm{Bandgap}\;\mathrm{energy}\;E_{g}$	eV	3.4	3.4
$\mathrm{Dielectric}\;\mathrm{constant}\;\varepsilon_{r}$	1	9.5	9.5
Density of states *N*	${\mathrm{cm}}^{-3}$	$2.24\times 10^{18}$	$2.51\times 10^{19}$
Mobility μ	${\mathrm{cm}}^{2}\cdot s^{-1}\cdot V^{-1}$	400	8
$\mathrm{Saturation}\;\mathrm{velocity}\;v_{sat}$	$\mathrm{cm}\cdot s^{-1}$	$1.9\times 10^{7}$	$1\times 10^{6}$
Effective mass (relative) m	1	0.222	0.989
$\mathrm{Auger}\;\mathrm{coefficient}\;C^{Auger}$	${\mathrm{cm}}^{6}\cdot s^{-1}$	$1\times 10^{-30}$	$1\times 10^{-30}$
$\mathrm{SRH}\;\mathrm{lifetime}\;\tau^{SRH}$	s	$1\times 10^{-9}$	$1\times 10^{-9}$
$\mathrm{Radiative}\;\mathrm{coefficient}\;C_{opt}$	${\mathrm{cm}}^{3}\cdot s^{-1}$	$1.2\times 10^{-8}$	$1.2\times 10^{-8}$
$\mathrm{BBT}\;\mathrm{coefficient}\;A_{BBT}$	$V^{-1}\cdot {\mathrm{cm}}^{-1}\cdot s^{-1}$	$3\times 10^{12}$	$3\times 10^{12}$
$\mathrm{BBT}\;\mathrm{coefficient}\;B_{BBT}$	$V\cdot {\mathrm{cm}}^{-1}$	$3.5\times 10^{7}$	$3.5\times 10^{7}$
$\mathrm{BBT}\;\mathrm{coefficient}\;\gamma_{BBT}$	1	2	2
Impact constants a	${\mathrm{cm}}^{-1}$	$4.48\times 10^{8}$	$7.13\times 10^{6}$
Impact constants β	$V\cdot {\mathrm{cm}}^{-1}$	$3.39\times 10^{7}$	$1.46\times 10^{7}$

## References

[1] Mu Y., Wang C., Ren J., Zhu Y. (2021). Evaluation and experimental comparisons of different photodetector receivers for visible light communication systems under typical scenarios. Opt. Eng..

[2] Sato K., Hosoda T., Watanabe Y., Wada S., Iriguchi Y., Makita K., Shono A., Shimizu J., Sakamoto K., Watanabe I. Record highest sensitivity of 28.0 dBm at 10 Gb/s achieved by newly developed extremely-compact superlattice-APD module with TIA-IC. Proceedings of the Optical Fiber Communication Conference and Exhibit.

[3] Hoon J., Minkyu C., Xu Z., Frank M., Nepomuk O., Shen S., Theeradetch D., Dupuis R. (2023). Ion-implanted Al0.6Ga0.4N deep-ultraviolet avalanche photodiodes. Appl. Phys. Lett..

[4] Lee H.H., Doo K.-H., Mun S.-G., Kim K., Lee J.H., Kang S.-K., Park H., Park N., Park H., Chung H.S. (2016). Real-time demonstration of QoS guaranteed 25-Gb/s PON prototype with Ethernet-PON MAC/PHY and cost-effective APD receivers for 100-Gb/s access networks. Opt. Express.

[5] Milovančev D., Brandl P., Jukić T., Steindl B. (2019). Optical wireless APD receivers in 0.35 µm HV CMOS technology with large detection area. Opt. Express.

[6] Gnauck A., Iannone P., van Veen D., Houtsma V. (2015). 4 × 40-Gb/s TWDM PON downstream transmission over 42 km and 64-way power split using optical duobinary signals and an APD-based receiver. Opt. Express.

[7] Sun W., Fu Y., Lu Z., Campbell J. (2013). Study of bandwidth enhancement and non-linear behavior in avalanche photodiodes under high power condition. J. Appl. Phys..

[8] Nada M., Hoshi T., Yamazaki H., Hashimoto T., Matsuzaki H. (2015). Linearity improvement of high-speed avalanche photodiodes using thin depleted absorber operating with higher order modulation format. Opt. Express.

[9] Lee C., Lin Y., Lin W. (2016). Analysis of temperature dependence of linearity for SiGe HBTs in the avalanche region using Volterra series. Microelectron. Reliab..

[10] Wang Y., Yu J., Chi N., Chang G. (2015). Experimental demonstration of 120-Gb/s nyquist PAM8-SCFDE for short-reach optical communication. IEEE Photon. J..

[11] Haško D., Kováč J., Uherek F., Škriniarová J., Jakabovič J., Peternai L. (2006). Avalanche photodiode with sectional InGaAsP/InP charge layer. Microelectron. J..

[12] Beckerwerth T., Behrends R., Ganzer F., Runge P., Schell M. (2022). Linearity Characteristics of Avalanche Photodiodes For InP Based PICs. IEEE J. Sel. Top. Quantum Electron..

[13] Akiba M., Fujiwara M., Sasaki M. (2005). Ultrahigh-sensitivity high-linearity photodetection system using a low-gain avalanche photodiode with an ultralow-noise readout circuit. Opt. Lett..

[14] Wu C., Zhang G., Jia J., Hu H., Wu F., Wang S., Guo D. (2024). Highly Polarization-Deep-Ultraviolet-Sensitive β-Ga_2_O_3_ Epitaxial Films by Disrupting Rotational Symmetry and Encrypted Solar-Blind Optical Communication Application. J. Phys. Chem. Lett..

[15] Xie F., Yang G., Zhou D., Lu H., Wang G. (2016). Frequency response and design consideration of GaN SAM avalanche photodiodes. Appl. Phys. A.

[16] Nada Y.Y.M., Matsuzaki H. (2017). A high-linearity avalanche photodiodes with a dual-carrier injection structure. IEEE Photonics Technol. Lett..

[17] Xing H., Zhang J., Liu A., Yang Y. (2019). Design of high linearity InGaAs/InP avalanche photodiode with a hybrid absorption layer structure. Infrared Phys. Technol..

[18] Li Y., Yuan W., Li K., Duan X., Liu K., Huang Y. (2022). InGaAs/InAlAs SAGCMCT avalanche photodiode with high linearity and wide dynamic range. Chin. Opt. Lett..

[19] Jiang Y., Chen J. (2019). Optimization of the Linearity of InGaAs/InAlAs SAGCM APDs. J. Light. Technol..

[20] Yang S., Zhou D., Xu W., Cai X., Lu H., Chen D., Ren F., Zhang R., Zheng Y. (2017). 4H-SiC Ultraviolet Avalanche Photodiodes with Small Gain Slope and Enhanced Fill Factor. IEEE Photonics J..

[21] Lee S., Jin X., Jung H., Lewis H., Liu Y., Guo B., Kodati S.H., Schwartz M., Grein C., Ronningen T.J. (2016). High gain, low noise 1550 nm GaAsSb/AlGaAsSb avalanche photodiodes. Optica.

[22] Guo S., Zhao X., He Y., Pan Z., Yang M., Cai Y., Fu X., Zhang L. (2022). High-Temperature Performance of the 4H-SiC n-p-n Bipolar UV Phototransistor. IEEE Sens. J..

[23] Wu L., Zhao D., Deng Y., Jiang D., Zhu J., Wang H., Liu Z., Zhang S., Zhang B., Yang H. (2012). Distribution of electric field and design of devices in GaN avalanche photodiodes. Sci. China Phys. Mech. Astron..

[24] Wang X., Hu W., Pan M., Hou L., Xu W.X., Li X., Chen X., Lu W. (2014). Study of gain and photoresponse characteristics for back-illuminated separate absorption and multiplication Gan avalanche photodiodes. J. Appl. Phys..

[25] Ji D., Chowdhury S. (2022). On the Scope of GaN-Based Avalanche Photodiodes for Various Ultraviolet-Based Applications. Front. Mater..

[26] Wang H., Xia H., Liu Y., Chen Y., Xie R., Wang Z., Wang P., Miao J., Wang F., Li T. (2024). Room-temperature low-threshold avalanche effect in stepwise van-der-Waals homojunction photodiodes. Nat. Commun..

[27] Carrano J.C., Lambert D.J.H., Eiting C.J., Collins C.J., Li T., Wang S., Yang B., Beck A.L., Dupuis R.D., Campbell J.C. (2000). GaN avalanche photodiodes. Appl. Phys. Lett..

[28] Pau J.L., McClintock R., Minder K., Bayram C., Kung P., Razeghi M., Muñoz E., Silversmith D. (2007). Geiger-mode operation of back-illuminated GaN avalanche photodiodes. Appl. Phys. Lett..

[29] Shockley W., Read W.T. (1952). Statistics of the Recombination of Holes and Electrons. Phys. Rev..

[30] Joyce W.B., Dixon R.W. (1977). Analytic Approximations for the Fermi Energy of an Ideal Fermi gas. Appl. Phys. Lett..

[31] Klaassen D.B.M., Slotboom J., Graaff H.D. (1992). Unified Apparent Bandgap Narrowing in n- and p-type Silicon. Solid-State Elect..

[32] Hurkx G.A.M., Graaf H.D., Klosterman W., Knuvers M. A Novel Compact Model Description of Reverse Biase Diode Characteristics including Tunneling. Proceedings of the ESSDERC’90: 20th European Solid State Device Research Conference.

[33] Overstraeten V., Deman H. (1970). Measurement of the Ionization Rates in Diffused Silicon p-n Junctions. Solid-State Electron..

[34] Keldysh L.V. (1960). Kinetic theory of impact ionization in semiconductors. Sov. Phys. JETP.

[35] Lina C., Jingshan W., Galen H., Hansheng Y., Roy S., Hoffman A.J., Patrick F. (2018). Experimental characterization of impact ionization coefficients for electrons and holes in GaN grown on bulk GaN substrates. Appl. Phys. Lett..

